# Growth and Adult Height in Patients with Crohn's Disease Treated with Anti-Tumor Necrosis Factor α Antibodies

**DOI:** 10.1371/journal.pone.0163126

**Published:** 2016-09-16

**Authors:** Sarah Bamberger, Christine Martinez Vinson, Damir Mohamed, Jérôme Viala, Jean-Claude Carel, Jean-Pierre Hugot, Dominique Simon

**Affiliations:** 1 Service de Gastroentérologie Pédiatrique, Assistance publique-Hôpitaux de Paris, Hôpital Robert Debré, Paris, France; 2 Unité d’Epidémiologie Clinique, Assistance publique-Hôpitaux de Paris, Hôpital Robert Debré, Paris, France; 3 Centre d’Investigation Clinique-Epidémiologie Clinique Unité 1426, Institut National de la Santé et de la Recherche Médicale (Inserm), Paris, France; 4 Service d'Endocrinologie Diabétologie Pédiatrique, Centre de Référence des Maladies Endocriniennes Rares de la Croissance, Assistance publique-Hôpitaux de Paris, Hôpital Robert Debré, Paris, France; 5 Université Paris Diderot, Sorbonne Paris Cité, Paris, France; 6 Institut National de la Santé et de la Recherche Médicale (Inserm), Unité 1141, DHU Protect, Paris, France; 7 Institut National de la Santé et de la Recherche Médicale (Inserm), Centre de Recherche sur l'Inflammation Unité 1149, Paris, France; University Hospital Llandough, UNITED KINGDOM

## Abstract

Inflammation contributes to growth failure associated with inflammatory bowel diseases. Anti-TNFα therapy induces sustained remission and short-term improvements in height velocity and/or height standard deviation score (H-SDS) patients with Crohn’s disease. The purpose of this study was to evaluate growth and adult height in patients with Crohn’s disease taking maintenance infliximab or adalimumab therapy.This university-hospital based retrospective study included 61 patients, with a median follow-up of 2.6 years (2.0; 3.3). 38 patients (62%) reached their adult height. H-SDS was collected at diagnosis and together with disease activity markers (Harvey-Bradshaw Index, albumin, and C-reactive protein) at treatment initiation (baseline), and follow-up completion. Wilcoxon’s signed-rank test was chosen for comparisons. Median H-SDS decreased from diagnosis to baseline (-0.08 [-0.73; +0.77] to -0.94 [-1.44; +0.11], p<0.0001) and then increased to follow-up completion (-0.63 [-1.08; 0.49], p = 0.003 versus baseline), concomitantly with an improvement in disease activity. Median adult H-SDS was within the normal range (-0.72 [-1.25; +0.42]) but did not differ from baseline H-SDS and was significantly lower than the target H-SDS (-0.09 [-0.67; +0.42], p = 0.01). Only 2 (6%) males had adult heights significantly below their target heights (10.5 and -13.5 cm [-1.75 and -2.25 SD]). In conclusion, anti-tumor necrosis factor α (TNF) therapy prevented loss of height without fully restoring the genetic growth potential in this group of patients with CD. Earlier treatment initiation might improve growth outcomes in these patients.

## Introduction

Growth failure is common in patients with childhood-onset Crohn’s disease (CD), both at diagnosis and during follow-up. Overall, about 20% of patients have a reduced adult height, defined as a greater than 2 SD loss of height versus height at disease onset or as a greater than 8 cm difference from target height [1, 2]. Thus, the treatment seeks not only to achieve disease remission, but also to optimize growth and pubertal development so that the adult height is within the target height range. The main causes of growth failure and pubertal delay are chronic inflammation, malnutrition, and prolonged corticosteroid therapy. Treatment options for obtaining a sustained disease remission include exclusive enteral nutrition, surgery, and nonsteroid immunosuppressive agents. In retrospective studies, these treatments improved growth in the short term (6–12 months). Significant catch-up growth has been reported after surgical resection of localized lesions before or during early puberty [3]. Exclusive enteral nutrition and azathioprine induce larger improvements in height velocity (HV) and height standard deviation score (H-SDS) compared to corticosteroid therapy [4, 5]. The effects of endocrine treatments on growth and puberty have also been evaluated in very small short-term studies. Testosterone for 6 months significantly improved growth and pubertal status in adolescents with inflammatory bowel disease (IBD) but its effects on adult height were not evaluated [6]. Therapeutical trials with recombinant human growth hormone in short children with IBD produced controversial results [7, 8] and have not been extended.

Anti-tumor necrosis factor α (anti-TNFα) therapy has dramatically modified the medical management of patients with CD. Among patients given biologics, 90% achieve a short-term remission and up to 60% experience sustained clinical benefits after 3 years of treatment [9]. Anti-TNFα antibodies have been reported to induce short-term improvements in HV and/or H-SDS [10, 11] but their effects on adult height are unknown.

Here, our aim was to evaluate the mid-term effects on growth of anti-TNF α maintenance therapy in children with CD, some of whom were followed until growth completion.

## Methods

### Ethics

This retrospective study was approved by the ethics committee of the Robert Debré Teaching Hospital, Paris, France, which waived the need for written informed consent (reference number: 2014/126, CNIL reference number 1763539). All study patients and/or their parents gave oral informed consent to study inclusion, which was noticed in patients ‘charts.

### Patients

We retrospectively reviewed the medical charts of children who received care for CD at the pediatric gastroenterology department of the Robert Debré Teaching Hospital, Paris, France, between January 1998 and January 2013. Inclusion criteria were CD meeting European Crohn’s and Colitis Organisation criteria [12] and anti-TNFα antibody therapy (infliximab or adalimumab) for at least 1 year. Exclusion criteria were episodic anti-TNFα antibody therapy, attainment of adult height before or during the first treatment year, and concomitant treatment with recombinant growth hormone (rhGH) or sex steroids (testosterone or estrogens, which may interfere with linear growth).

### Data collection

#### Auxologic parameters

Height (in cm) of parents and height (in cm) and weight (in kg) of patients 1 year before anti-TNFα initiation, at anti-TNFα initiation (baseline), and once a year thereafter were abstracted from the medical records. Body mass index (BMI, kg/m^2^) was computed as (weight in kg)/ (height in m) ^2^ and target height (cm) as ([father’s height in cm + mother’s height in cm]/2) + 6.5-cm for boys and -6.5-cm for girls. HV (cm/year) over a time interval as close as possible to 12 months (4 to 15 months) was computed as follows: ([height difference between the two time points, in cm]/months between the two time points)x12. Height, weight, BMI, and HV were expressed as standard deviation scores (SDS) over chronological age (CA) based on reference values for the French population [13, 14]. Bone age was assessed on radiographs of the left hand according to Greulich and Pyle [15]. Adult height was defined by a HV < 2 cm/year and/or bone age >15 years in girls and >17 years in boys. Adult height and target height were expressed as H-SDS based on reference values for adult height in the French population [14] (S1 File).

#### Disease parameters

The Paris classification was used to categorize the CD phenotype [16]. We recorded disease activity as evaluated clinically at each visit based on the Harvey-Bradshaw Index [17] and number of flares between consecutive visits. Harvey Bradshaw index categories were as follows: <4 inactive disease; ≥ 4 and <12 moderately active; ≥ 12 highly active. A flare was defined as hospital admission or a change in treatment. Plasmatic C Reactive Protein (CRP, mg/l; normal<10mg/L) and albumin (g/l; normal: 40-51g/L) were recorded (S1 File).

Anti TNFα therapy was initiated as the first-line therapy in patients with perianal disease. In other patients, the first-line treatment was a corticosteroid or another immunosuppressive drug (azathioprine, 6 mercaptopurin, methotrexate or tacrolimus); if appropriate, an anti-TNFα was used as the second-line treatment. Induction and maintenance doses were those recommended in guidelines for anti-TNFα therapy [18]: infliximab, 5mg/kg at weeks 0, 2 and 6 and then 5mg/kg injection every 8 weeks; adalimumab, 160 mg if weight >40kg or 80 mg if weight <40kg at week 0, 80mg if weight >40kg or 40 mg if weight <40kg at week 2 and thereafter a subcutaneous injection every other week of 40 mg if weight >40kg or 20mg if weight <40kg. When disease control was inadequate, the dosage was increased or the dosing interval decreased. Infliximab was used first. Patients with an anaphylactic reaction to infliximab were switched to adalimumab.

### Statistical analysis

Data were described as median, interquartile range (25^th^-75^th^ percentiles), range (minimum-maximum) or number (%) as appropriate. As the number of patients decreased during follow-up, changes in auxologic parameters in the 61 included patients are reported for three time points: diagnosis, anti-TNFα initiation (baseline) and last follow-up. H-SDS, HV-SDS and BMI-SDS values over time were compared using Wilcoxon’s signed rank test. Bonferroni’s correction for multiple comparisons was applied when appropriate: this procedure set the threshold for significance at 0.0125 for H-SDS and 0.025 for BMI-SDS, CRP, albumin. At anti-TNFα initiation, auxologic parameters and disease characteristics were compared between the patients who attained their adult height and those who were still growing, using the nonparametric Mann-Whitney test for continuous variables. For categorical variables, we used the chi-square test, or Fischer’s exact test as appropriate. These tests were two-sided tests; *P*-values less than 0.05 were considered significant. All statistical analyses were performed using SAS software, v. 9.4 (SAS Institute Inc., Cary, NC).

## Results

### Patients

**Fig 1:**
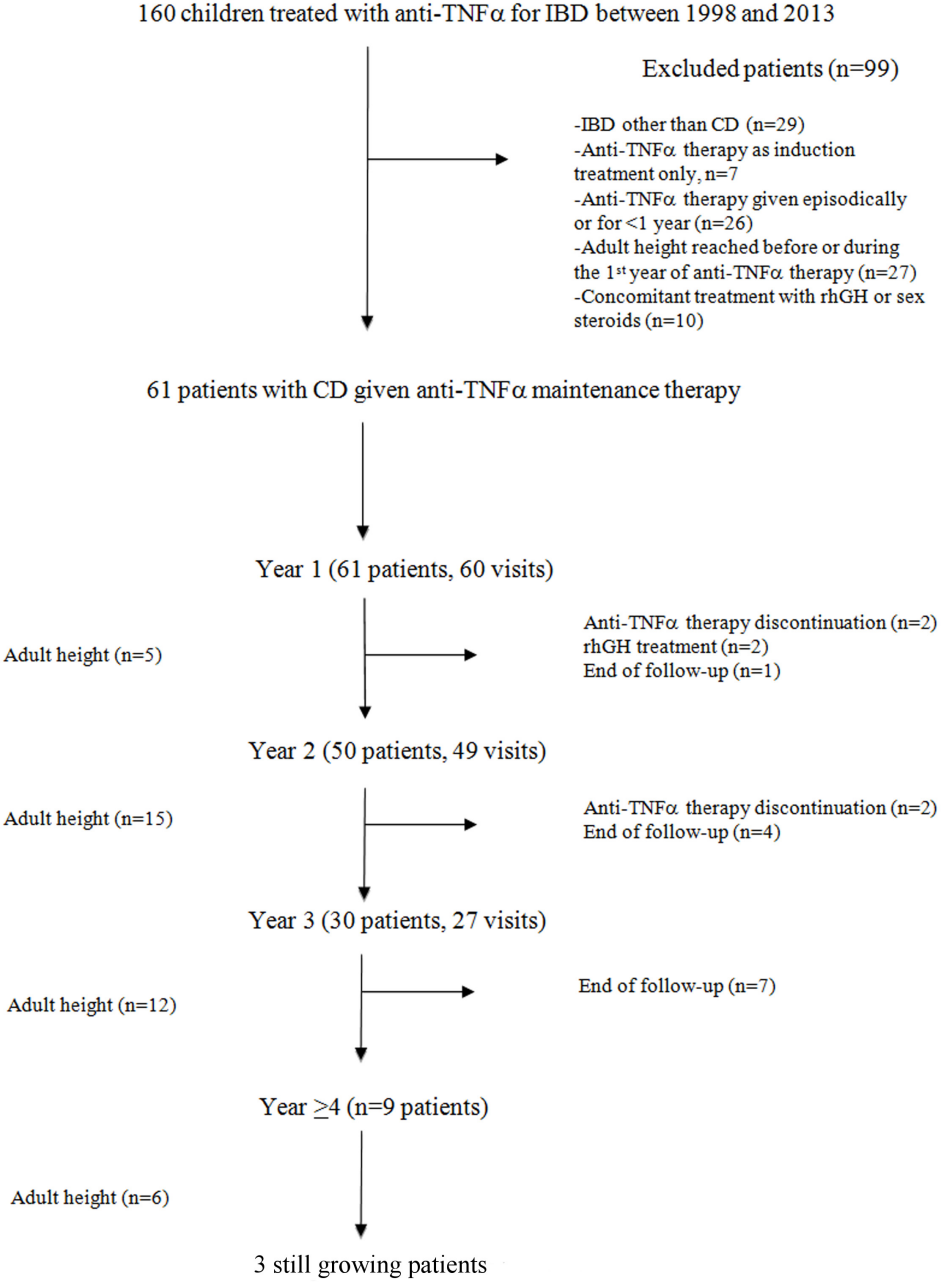
Patient flow chart. The number of patients and visits at each year of the study are reported into brackets. Five patients missed their visit of follow-up at Year 1 (n = 1),Year 2 (n = 1) and Year 3 (n = 3).

Of the 160 patients given anti-TNFα antibodies for IBD during the study period, 61 (38 males) met the inclusion and exclusion criteria, as shown in the patient flow chart (Fig 1). Table 1 lists the main patient characteristics before anti-TNFα initiation. Eight patients (13%) had no treatment. Other patients received either one treatment (n = 16, 26%) or two (n = 30, 49%) or three (n = 7, 11%) combined therapies (S1 Table). Specific reasons for starting anti-TNFα therapy were relapses despite immunosuppressive therapy (n = 38, 62%), corticosteroid dependence (n = 25, 41%), perianal disease (n = 10, 16%), and contraindications to immunosuppressive drugs (n = 9, 15%). Twenty-one patients had two reasons for initiating anti-TNFα therapy (S2 Table). Median follow-up duration during maintenance treatment was 2.6 (2.0; 3.3) years, (range: 1–7.6). The first anti-TNFα used was infliximab in all patients. Subsequently, 11 patients were switched to adalimumab because of incomplete disease control (n = 10) or an anaphylaxis-like reaction to infliximab (n = 1). One patient with incomplete responses to infliximab then adalimumab has been switched to certolizumab

**Table 1:** Patient characteristics at anti-TNFα initiation (baseline).

	Patients (n = 61)	Males (n = 38)	Females (n = 23)	*P* value
Age (y)	13.3 (12.2; 14.7)	13.5 (12.4; 14.9)	13.1 (11.4; 13.9)	0.23
Disease duration (y)	1.9 (1.2; 2.9)	2.1 (1.1; 2.9)	1.9 (1.3; 2.5)	0.84
Disease location, n (%)				
*L3or L1+L4 or L3+L4*	50 (82%)			
*L2 or L2+L4*	11 (18%)			
Disease behavior, n (%)				
*B1*	50 (82%)			
*B2*	1 (2%)			
*B1+B2*	10 (16%)			
Perianal disease, n (%) n = 60	27(45%)			
Extradigestive symptoms, n (%)	20 (33%)			
Harvey Bradshaw Index,n (%) n = 60				
*[0–4[*	16(27%)			
*[4–12[*	41(68%)			
*≥12*	3(5%)			
Current therapy, n (%)*				
*No therapy*	8(13%)			
*5 aminosalicylates*	8 (13%)			
*Corticosteroids*	32 (53%)			
*Budesonide*	6 (10%)			
*Immunomodulator*	43 (70%)			
*Enteral nutrition*	8 (13%)			
HV- SDS/CA (n = 60)	-1.5 (-2.5; -0.6)	-1.4 (-2.4; -0.6)	-1.7 (-2.8; -0.6)	0.81
HV cm/y (n = 60)	3.1 (1.7; 5.1)	3.4 (1.6; 5.2)	2.9 (1.7; 4.8)	
H-SDS /CA (n = 60)	-0.94 (-1.44; 0.11)	-0.89 (-1.35; 0.09)	-1.00 (-1.76; 0.54)	0.69
BMI-SDS /CA (n = 60)	-0.58 (-1.32; 0.32)	-0.69 (-1.58; -0.04)	-0.36 (-0.94; 0.52)	0.12
Bone age (y) (n = 40)	12.8 (11.3; 14.0)	13.0 (11.6; 14.0)	12.3 (10.2; 13.5)	

L1, ileal; L2, colonic; L3, ileocolonic; L4, isolated upper disease; B1, non-structuring, non-penetrating; B2, structuring.

n = 61, if not specified.

* Some patients received combined treatments explaining that the number of patients exceeds 61 (S1 Table)

### Disease activity during follow up

During anti-TNFα treatment, the percentage of patients with moderate or severe disease according to the Harvey Bradshaw index decreased from 72% at baseline to 13% at last follow-up. At last follow-up, 53 (87%) patients had inactive disease and no patient had a severe disease. Of the 32 patients on corticosteroid therapy at baseline, 27 were off this treatment at last follow-up. The percentage of patients on immunosuppressive drugs fell from 70% at baseline to 33% at last follow-up. Median plasma CRP decreased significantly from 30.0 mg/L (7.0; 55.5) at baseline to 7.0 mg/L (7.0; 17.5) after the first year of anti-TNFα (p<0.001) and then remained stable (7.0 mg/L[7.0; 11.0] at last follow-up; p<0.001vs baseline). Median plasma albumin improved significantly from 32.8 g/L (28.0; 37.0) at baseline to 38.7 g/L (34.0; 42.5), after the first year of treatment (p<0.001) then levelled off (41.2 g/L [36.3; 42.9] at last follow-up, p<0.001 vs baseline).

### Linear growth during follow-up

**Fig 2:**
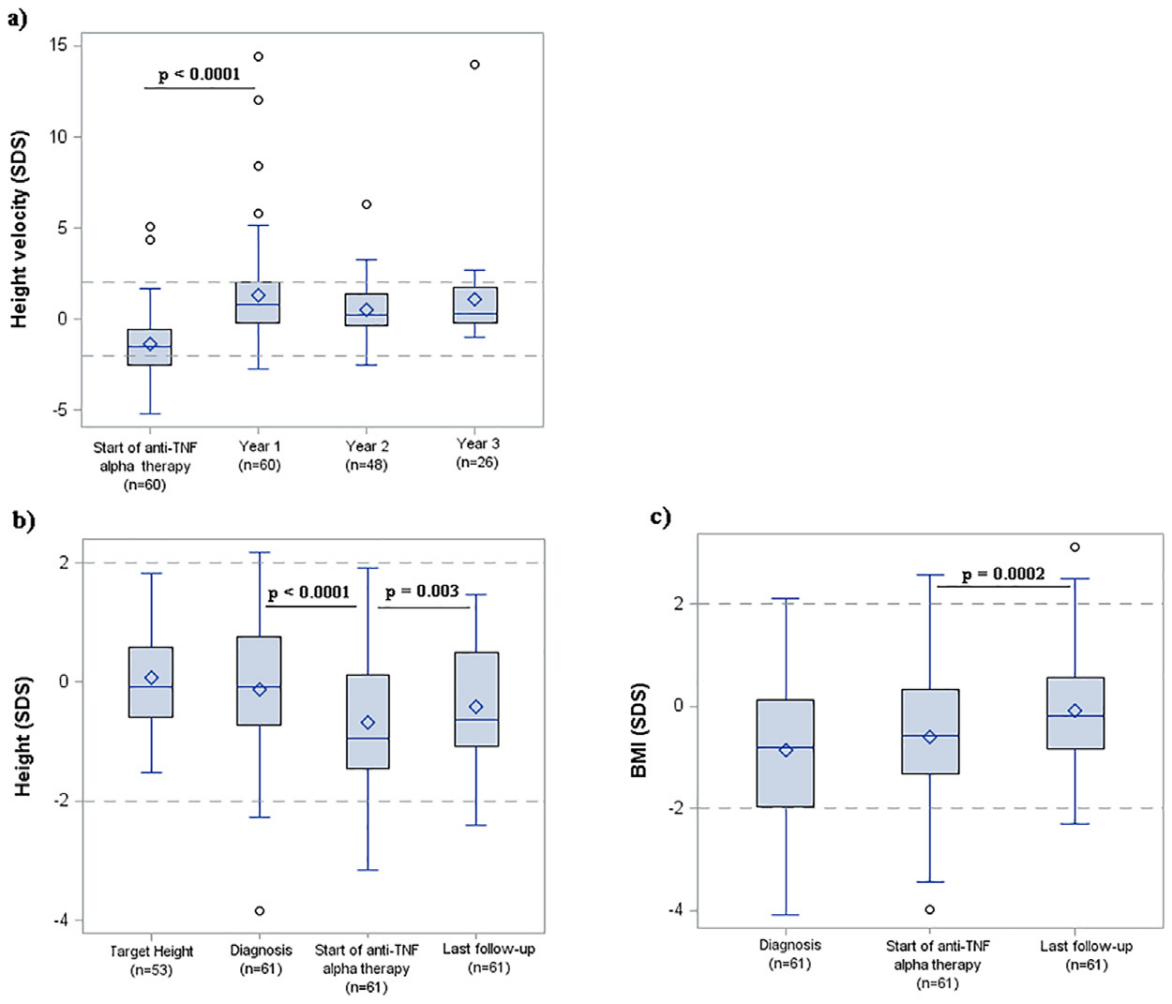
**(a) Changes in HV-SDS/CA during the first 3 years of follow-up. (b) Changes in H-SDS and in (c) BMI-SDS from diagnosis to anti-TNFα initiation to last follow-up.** Box plots show median values, 25^th^ -75^th^ percentiles, and range. Dots in the box plots represent mean values. The number of patients at each follow-up time point is reported in brackets. Dashed lines represent mean, -2 SD and +2 SD values.

Median HV significantly increased in the first year of treatment from -1.5 SDS (-2.5; -0.6) to +0.8 SDS (-0.2; +2.1) (p<0.0001) and then remained stable during the following 3 years (Fig 2a).

At diagnosis, median H-SDS was in the normal range (-0.08 [-0.73; +0.77]) and did not differ from the median target H-SDS (-0.08 [-0.58; +0.58], p = 0.84). From diagnosis to anti-TNFα initiation, median H-SDS decreased significantly to -0.94 (-1.44; +0.11) (p<0.0001 vs H-SDS at diagnosis), which was equivalent to a height loss of -0.56 SD (-1.07; -0.06). During treatment, there was a height gain of +0.26 SD (-0.02; +0.76) and a significant improvement in H-SDS to -0.63 (-1.08; 0.49) at last follow-up (p = 0.003 vs baseline) (Fig 2b). BMI-SDS remained stable from diagnosis (-0.81 [-1.96; +0.11]) to anti-TNFα initiation (-0.58 [-1.32; + 0.32], p = 0.13) then increased significantly to -0.18 (-0.84; 0.56) at last follow-up (p = 0.0002 vs baseline) (Fig 2c).

Adult height was achieved by 38 (62%) patients, 21 boys and 17 girls after a median treatment duration of 2.6 years ([2; 3.3]; range: 1.1; 5.5), at a median CA of 16.9 years (16; 17.8). Table 2 lists their main characteristics. At anti-TNF initiation, these 38 patients were older (14.0 years [13.0; 15.1]) than the 23 patients who were still growing (12.1 years [10.7; 13.3], p = 0.0001). The disease characteristics and the auxologic parameters (except bone age) did not differ between these 2 groups (Table 3). In the group of 38 patients, H-SDS at anti-TNFα initiation was lower than at diagnosis and no significant catch-up growth occured. Their adult H-SDS (-0.72 [-1.25; +0.42]; range: -2.41; 1.47) was in the normal range in both sexes but lower than the target H-SDS (-0.09 [-0.67; +0.42], p = 0.01), the median difference being -2.5 cm (-5.0; 0) in girls and -2.0 cm (-6.0; 2.5) in boys. Of 2 patients (2 boys, 6% of the group) among the 32 patients whose target height was available, 2 (6%, both male) had a severe height deficiency (-10.5cm [-1.75 SD] and -13.5cm [-2.25 SD] vs target height, respectively). Surprisingly, the patient who had been switched to Certolizumab did not exhibit a severe height deficiency, his adult height being only 1.7 cm (-0.28 SD) below his target height.

**Table 2:** Main characteristics of the 38 patients who attained their adult height during follow-up.

	Diagnosis	Anti-TNFα initiation	At adult height
Chronological age (y)	11.7 (10.5; 13.6)	14.0 (13.0; 15.1)	16.9 (16; 17.8)
Disease duration (y)		1.95 (1.30; 2.90)	
Harvey-Bradshaw Index, n (%)	NA		
*[0–4[*		9 (24%)	32 (84%)
*[4–12[*		26 (68%)	6 (16%)
*≥12*		3 (8%)	0 (0%)
Current therapy^#^, n (%)			
*No therapy*		4 (11%)	
*5 aminosalicylates*	27 (71%)	3 (8%)	2 (5%)
*Corticosteroids*	33 (87%)	21 (55%)	3 (8%)
*Budesonide*	12 (32%)	4 (11%)	0
*Immunomodulator*	34 (89%)	30 (79%)	8 (21%)
*Enteral nutrition*	21 (55%)	5 (13%)	2 (5%)
CRP (mg/L)	NA	28 (12; 49)	7 (7; 11)
Albumin (g/L)	NA	34.4 (31.0; 37.8)	41.3 (36.6; 43.0)
Bone age (y) in girls *(n = 10)*		12.8 (11.0; 14.0)	
Bone age(y) in boys *(n = 17)*		14.0 (13.0; 14.0)	
Target H-SDS *(n = 32*)	-0.09 (-0.67; 0.42)		
Target height (cm)			
*Girls (n = 13/17)*	163.0 (160.5; 165.0)		
*Boys(n = 19/21)*	175.0 (171.5; 178.5)		
H-SDS	-0.06 (-0.73; 0.77)	-0.95* (-1.4; 0.5)	-0.72** (-1.25; 0.42)
*Girls*	-0.08 (-1.55; 0.77)		-0.63 (-1.34; 0.27)
*Boys*	-0.04 (-0.63; 0.77)		-0.92 (-1.17; 0.42)
Height (cm) in girls	144 (135; 146)		160 (156; 165)
Height (cm) in boys	147 (140; 157)		170 (168.5; 178)
BMI-SDS	-0.98 (-2.32; 0.11)	-0.62 (-1.62; 0.26)	-0.09*** (-0.79; 0.27)

n = 38 (17 girls, 21 boys) if not specified.

*P = 0.0002 versus height at diagnosis,

**P = 0.01 versus target height;

***P = 0.0002 versus BMI at Anti-TNFα initiation.

^#^ Some patients received combined therapy, explaining that the number of patients exceeds 38.

**Table 3:** Comparisons between the 23 patients who were still growing and the 38 patients who attained their final height at the end of follow-up.

Anti-TNFα initiation	Still growing patients (n = 23)	Patients at final height (n = 38)	P value
Chronological age (y)	12.1 (10.7; 13.3)	14.0 (13.0; 15.1)	0.0001
Disease duration (y)	1.9 (1.1; 2.8)	1.95 (1.3; 2.9)	0.59
Disease location, n (%)			0.92
*L3or L1+L4 or L3+L4*	19 (83%)	31 (82%)	
*L2 or L2+L4*	4 (17%)	7 (18%)	
Disease behavior, n (%)			0.83
*B1*	20 (87%)	30 (79%)	
*B2*	0 (0%)	1 (3%)	
*B1+B2*	3 (13%)	7 (18%)	
Perianal disease, n (%)	3 (13%)	7/38 (18%)	0.73
Extradigestive symptoms, n (%)	9 (39%)	11/38 (29%)	0.41
Harvey-Bradshaw Index, n (%)			0.89
*[0–4[*	7 (30%)	9 (24%)	
*[4–12[*	15 (65%)	26 (68%)	
*≥12*	1 (4%)	3 (8%)	
Current therapy, n (%)			
*No therapy*	4 (17%)	4(11%)	
*5 aminosalicylates*	5 (22%)	3 (8%)	0.14
*Corticosteroids*	11 (48%)	21 (55%)	0.57
*Budesonide*	2 (9%)	4 (11%)	
*Immunomodulator*	13 (57%)	30 (79%)	0.08
*Enteral nutrition*	3 (13%)	5 (13%)	
CRP (mg/L)	39.0 (7.0; 78.0)	28.0 (12.0; 49.0)	0.59
Albumin (g/L)	29.0 (26.0; 35.8)	34.4 (31.0; 37.8)	0.09
Bone age (y)	11.5 (10.2; 12.5) *n = 13*	14.0 (12.3; 14.0) *n = 27*	0.002
Target H-SDS	0.33 (-0.53;1.08) *n = 21*	-0.09 (-0.67; 0.42) *n = 32*	0.2
H-SDS	-0.86 (-1.66; 0.08)	-0.95 (-1.40; 0.50)	0.96
BMI-SDS	-0.49 (-1.24; 0.32)	-0.62 (-1.62; 0.26)	0.34

n = 23 or 38, if not specified.

## Discussion

We describe linear growth in children with anti-TNFα antibodies. This treatment stopped the height loss observed after diagnosis, stabilizing the H-SDS. Three-fifths of patients were followed until they reached their adult height. Catch-up growth occurred during treatment in the overall population. However, adult height, although within the normal range, was not improved compared to height at anti-TNFα initiation and was significantly lower than the target height. However, a significant reduction in adult height was very uncommon (2/32 patients with available data).

Inflammation and malnutrition contribute to growth failure associated with IBD. In a prepubertal rat model of colitis with plasma IL-6 elevation, a comparison with healthy rats fed with the same diet to eliminate differences due to undernutrition showed that inflammation explained 30%-40% of growth retardation [19]. Animals with colitis also exhibit pubertal delay which can be partially corrected by anti-cytokine treatment [20, 21] Growth-retarded children and adolescents with CD have a variety of endocrine profiles ranging from growth hormone resistance to growth hormone deficiency [22, 23] which reflect the impact on the somatotropic axis of cytokines, under-nutrition and sex-steroid deficiency. Furthermore, the deleterious effects of cytokines and undernutrition on the growth plate [24] contribute to slow linear growth. Thus, optimal nutritional support and tight inflammation control are major treatment goals in children with CD.

At diagnosis, BMI was normal and linear growth was within the target channel. Subsequently, however, significant height loss occurred over the years preceding anti-TNFα initiation. The clinical implication is that HV must be monitored closely to ensure that anti-TNFα treatment is started before severe growth failure develops. Inadequate inflammation control is the probable source of the height loss, given our findings regarding the disease activity markers. Furthermore, uncontrolled inflammation despite conventional treatment was the main reason for starting anti-TNFα therapy.

We chose Harvey Bradshaw index and plasma levels of CRP and albumin to monitor the effectiveness of anti-TNFα therapy because these parameters have been reported to be sensitive markers for inflammation control in everyday clinical practice [17, 25]. Anti-TNFα was associated with significantly better disease control: the Harvey-Bradshaw index improved, most patients were able to discontinue corticosteroid therapy, the CRP levels returned to normal and the albumin levels showed a sustained increase at last follow-up. Concomitantly, HV increased during the first treatment year and H-SDS improved significantly. These results support those previously reported and underline the link between linear growth and inflammation during biologic therapy. In 78 children with CD, larger decreases in TNFα and IL-6 levels during infliximab therapy were associated with larger increases in H-SDS [26]. Other studies found improvements in growth outcomes confined to those patients who achieved a remission during treatment [10, 11, 27–31].

In our patients, anti-TNFα was started late (about 2 years) after the diagnosis. The height loss that occurred after the diagnosis and the catch-up growth after anti-TNFα initiation suggest that anti-TNFα therapy might deserve to be started earlier during the course of the disease. A post hoc propensity-score analysis of observational data showed that starting anti-TNFα therapy within 3 months after the diagnosis was associated with a significant H-SDS gain during the first year treatment whereas H-SDS remained unchanged in patients treated early with another immunomodulator and in those given no early immunotherapy[32].

Of our 61 patients, 38(62%) attained their adult height under anti-TNFα therapy. Their adult H-SDS was within the normal range, but remained significantly lower than their target height, indicating that anti-TNFα treatment failed to fully restore their genetic growth potential. However, adult height is within ± 1.5 SDS of target H in 95% of normal children [33]. In our study, only 2 boys (6% of the group that attained adult height) were outside this range, i.e., had severe height deficiency.

The efficacy of anti-TNFα therapy in pediatric patients with CD has been well established. Consequently, a randomized controlled comparison of patients with or without anti-TNFα therapy would have been unethical. Instead, we compared growth before and after anti-TNFα initiation. We also compared adult height in our study to that reported before the introduction of anti-TNFα therapy. A study reported in 1993 found that 12% IBD patients had a reduced adult height defined as a height loss of 2SD or more from disease onset [2]. In 123 patients with CD studied after height completion, adult height was within the normal range overall but was >8 cm below the target height in 19% of patients [1]. In 135 patients with CD, adult H-SDS was not significantly different from the target height but was significantly lower in patients with prepubertal vs pubertal disease [34]. In our study, the percentage of patients with a significant growth deficiency after growth completion was very small, but the number of patients having reached their adult height was too small to allow definite conclusions about the effects of biotherapy on adult height.

Patients who attained their adult height did not experience catch-up growth after anti-TNFα initiation. In our patients, anti-TNFα therapy was started fairly late. Bone age was not delayed and was consistent with on-going puberty. In previous studies, a key factor in the growth-promoting effects of anti-TNFα therapy was the stage of puberty at treatment initiation: the gains of height were greater in patients treated in early than late puberty [27, 30]. Then, the failure to obtain catch-up growth was probably due to the limited remaining growth potential at treatment initiation. This point supports early anti-TNFα initiation. However, we cannot exclude that suboptimal growth occurred during puberty and negatively affected adult height. A limitation of our study is the absence of data on puberty progression and on the magnitude and duration of pubertal growth. Eventually, we cannot rule out that the absence of catch-up growth reflected discontinuous control of inflammation as the markers of inflammation were evaluated at only at 3 time points. Long-term anti-TNFα antibodies were effective in most studies. However, subclinical reactivation of the inflammatory process between infusions or escape phenomenon can occur over time [30, 35] limiting the beneficial effects of treatment on growth.

## Conclusion

This study showed that patients with pediatric-onset CD experienced a significant height loss within the first few years after the diagnosis, with a risk of failing to achieve the target height. Anti-TNFα antibodies can prevent further loss of height during the course of the disease, while ensuring remission of the inflammatory process. Our patients reached a normal adult height but failed to achieve their full growth potential. Optimization of anti-TNFα therapy, particularly via earlier initiation, may improve growth outcomes. Further studies with larger numbers of patients having achieved their adult height are needed to assess the impact of anti-TNFα therapy on linear growth in pediatric patients with CD.

## Supporting Information

S1 File List of variables. (DOCX)

S1 Table Patients’ treatments at anti-TNFα initiation. (DOCX)

S2 Table Reasons for starting anti-TNF α therapy. (DOCX)

## References

[1] Sawczenko A, Ballinger AB, Savage MO, Sanderson IR. Clinical features affecting final adult height in patients with pediatric-onset Crohn's disease. Pediatrics 2006;11:124–9. 10.1542/peds.2005-2931 16818557

[2] Markowitz J, Grancher K, Rosa J, Aiges H, Daum F. Growth failure in pediatric inflammatory bowel disease. J Pediatric Gastroenterol Nutrition 1993;16:373–80. 10.1097/00005176-199305000-00005 8315544

[3] Lipson AB, Savage MO, Davies PS, Bassett K, Shand WS, Walker-Smith JA l. Acceleration of linear growth following intestinal resection for Crohn disease. Eur J Pediatr 1990;149:687–90. 2209659 10.1007/BF01959522

[4] Sanderson IR, Udeen S, Davies PS, Savage MO, Walker-Smith JA. Remission induced by an elemental diet in small bowel Crohn's disease. Arch Dis Child 1987;62:123–7. 3548602 10.1136/adc.62.2.123 PMC1778272

[5] Fuentes D, Torrente F, Keady S, Thirrupathy K, Thomson M, Walker-Smith JA, et al High-dose azathioprine in children with inflammatory bowel disease. Aliment Pharmacol Ther 2003;17:913–21. 12656694 10.1046/j.1365-2036.2003.01540.x

[6] Mason A, Wong SC, McGrogan P, Ahmed SF. Effect of testosterone therapy for delayed growth and puberty in boys with inflammatory bowel disease. Horm Res Paediatr 2011;75:8–13. 10.1159/000315902 20664179

[7] Mauras N, George D, Evans J, Milov D, Abrams S, Rini A, et al Growth hormone has anabolic effects in glucocorticosteroid-dependent children with inflammatory bowel disease: a pilot study. Metabolism 2002;51:127–35. 11782884 10.1053/meta.2002.28972

[8] Calenda KA, Schornagel IL, Sadeghi-Nejad A, Grand RJ. Effect of recombinant growth hormone treatment on children with Crohn's disease and short stature: a pilot study. Inflamm Bowel Dis 2005;11:435–41. 15867582 10.1097/01.mib.0000159321.58773.a6

[9] Hyams J, Walters TD, Crandall W, Kugathasan S, Griffiths A, Blank M, et al Safety and efficacy of maintenance infliximab therapy for moderate-to-severe Crohn's disease in children: REACH open-label extension. Cur Medical Res Opin 2011;27:651–62. 10.1185/03007995.2010.547575 21241207

[10] Malik S, Ahmed SF, Wilson ML, Shah N, Loganathan S, Naik S, et al The effects of anti-TNF-alpha treatment with adalimumab on growth in children with Crohn's disease (CD). J Crohn's Colitis 2012;6:337–44. 22405171 10.1016/j.crohns.2011.09.004

[11] Borrelli O, Bascietto C, Viola F, Bueno de Mesquita M, Barbato M, Mancini V, et al Infliximab heals intestinal inflammatory lesions and restores growth in children with Crohn's disease. Dig Liver Dis 2004;36:342–7. 15191204 10.1016/j.dld.2003.12.014

[12] Travis SP, Stange EF, Lemann M, Oresland T, Chowers Y, Forbes A, et al European evidence based consensus on the diagnosis and management of Crohn's disease: current management. Gut 2006;55 Suppl 1:i16–35. 16481629 10.1136/gut.2005.081950b PMC1859997

[13] Rolland-Cachera MF, Cole TJ, Sempe M, Tichet J, Rossignol C, Charraud A. Body Mass Index variations: centiles from birth to 87 years. Eur J Clin Nutrition 1991;45:13–21. 1855495

[14] Sempe M, Pedron G. Auxologie méthode et séquences. Paris: Théraplix;1979.

[15] Greulich WW, Pyle SY. Radiographic atlas of skeletal development of the hand and wrist.2nd ed Stanford: Stanford University Press; 1959.

[16] Levine A, Griffiths A, Markowitz J, Wilson DC, Turner D, Russell RK, et al Pediatric modification of the Montreal classification for inflammatory bowel disease: the Paris classification. Inflamm Bowel Dis 2011;17:1314–21. 10.1002/ibd.21493 21560194

[17] Harvey RF, Bradshaw JM. A simple index of Crohn's-disease activity. Lancet 1980;1:514 6102236 10.1016/s0140-6736(80)92767-1

[18] Ruemmele FM, Veres G, Kolho KL, Griffiths A, Levine A, Escher J, et al Consensus guidelines of ECCO/ESPGHAN on the medical management of pediatric Crohn's disease. J Crohns Colitis 2014;8: 1179–207. 10.1016/j.crohns.2014.04.005 24909831

[19] Ballinger AB, Azooz O, El-Haj T, Poole S, Farthing MJ. Growth failure occurs through a decrease in insulin-like growth factor 1 which is independent of undernutrition in a rat model of colitis. Gut 2000;46:694–700. 10764714 10.1136/gut.46.5.695 PMC1727919

[20] DeBoer MD, Li Y, Cohn S. Colitis causes delay in puberty in female mice out of proportion to changes in leptin and corticosterone. J Gastroenterol 2010;45:277–84. 10.1007/s00535-009-0192-x 20072791 PMC2850610

[21] Deboer MD, Steinman J, Li Y. Partial normalization of pubertal timing in female mice with DSS colitis treated with anti-TNF-alpha antibody. J Gastroenterol 2012;47:647–54. 10.1007/s00535-012-0542-y 22322660 PMC3378759

[22] MacRae VE, Wong SC, Farquharson C, Ahmed SF. Cytokine actions in growth disorders associated with pediatric chronic inflammatory diseases (review). Int J Mol Med 2006;18:1011–8. 17089003 10.3892/ijmm.18.6.1011

[23] Wong SC, Smyth A, McNeill E, Galloway PJ, Hassan K, McGrogan P, et al The growth hormone insulin-like growth factor 1 axis in children and adolescents with inflammatory bowel disease and growth retardation. Clin Endocrinol 2010;73:220–8. 10.1111/j.1365-2265.2010.03799.x 20184596

[24] Martensson K, Chrysis D, Savendahl L. Interleukin-1beta and TNF-alpha act in synergy to inhibit longitudinal growth in fetal rat metatarsal bones. J Bone Miner Res 2004;19:1805–12. 15476580 10.1359/JBMR.040805

[25] Lonnkvist MH, Theodorsson E, Holst M, Ljung T, Hellstrom PM. Blood chemistry markers for evaluation of inflammatory activity in Crohn's disease during infliximab therapy. Scand J Gastroenterol 2011;46:420–7. 10.3109/00365521.2010.539253 21114432

[26] Thayu M, Denson LA, Shults J, Zemel BS, Burnham JM, Baldassano RN, et al Determinants of changes in linear growth and body composition in incident pediatric Crohn's disease. Gastroenterology 2010;139:430–8. 10.1053/j.gastro.2010.04.044 20417635 PMC2910790

[27] Walters TD, Gilman AR, Griffiths AM. Linear growth improves during infliximab therapy in children with chronically active severe Crohn's disease. Inflamm Bowel Dis 2007;13:424–30. 17206672 10.1002/ibd.20069

[28] Crombe V, Salleron J, Savoye G, Dupas JL, Vernier-Massouille G, Lerebours E, et al Long-term outcome of treatment with infliximab in pediatric-onset Crohn's disease: a population-based study. Inflamm Bowel Dis 2011; 2144–52. 10.1002/ibd.21615 21287665

[29] Hyams JS, Griffiths A, Markowitz J, Baldassano RN, Faubion WA Jr., Colletti RB, et al Safety and efficacy of adalimumab for moderate to severe Crohn's disease in children. Gastroenterology 2012;143:365–74. 10.1053/j.gastro.2012.04.046 22562021

[30] Church PC, Guan J, Walters TD, Frost K, Assa A, Muise AM, et al Infliximab maintains durable response and facilitates catch-up growth in luminal pediatric Crohn's disease. Inflamm Bowel Dis 2014;20:1177–86. 10.1097/MIB.0000000000000083 24865777

[31] Assa A, Hartman C, Weiss B, Broide E, Rosenbach Y, Zevit N, et al Long-term outcome of tumor necrosis factor alpha antagonist's treatment in pediatric Crohn's disease. J of Crohns Colitis 2013;7:369–76. 22483567 10.1016/j.crohns.2012.03.006

[32] Walters TD, Kim MO, Denson LA, Griffiths AM, Dubinsky M, Markowitz J. et al Increased effectiveness of early therapy with anti-tumor necrosis factor-alpha vs an immunomodulator in children with Crohn's disease. Gastroenterology 2014;146:383–91. 10.1053/j.gastro.2013.10.027 24162032

[33] Luo ZC, Albertsson-Wikland K, Karlberg J. Target height as predicted by parental heights in a population-based study. Pediatr Res 1998;44:563–71. 9773847 10.1203/00006450-199810000-00016

[34] Alemzadeh N, Rekers-Mombarg LT, Mearin ML, Wit JM, Lamers CB, van Hogezand RA. Adult height in patients with early onset of Crohn's disease. Gut 2002;51:26–9. 12077087 10.1136/gut.51.1.26 PMC1773272

[35] Vespasiani Gentilucci U, Caviglia R, Picardi A, Carotti S, Ribolsi M, Galati G, et al Infliximab reverses growth hormone resistance associated with inflammatory bowel disease. Aliment Pharmacol Ther 2005;21:1063–71. 15854167 10.1111/j.1365-2036.2005.02449.x

