# Supplementary material for: Growth and Adult Height in Patients with Crohn's Disease Treated with Anti-Tumor Necrosis Factor α Antibodies
Source: PLoS One. 2016 Sep 16;11(9):e0163126. doi: 10.1371/journal.pone.0163126 (PMC5026336; doi:10.1371/journal.pone.0163126)
Supplement: S1 File — (DOCX) [file pone.0163126.s001.docx]

Réf : CAV150V01

**BIOCROHN**

**Impact des biothérapies sur la croissance staturale au long cours des enfants traités pour une maladie de Crohn**

Investigateur principal : Dominique SIMON

Autres référents : Christine MARTINEZ VINSON

Sarah BAMBERGER

Promoteur : AP-HP

| Damir MOHAMED  Data manager | Pr. Corinne ALBERTI  Responsable de l’UEC |
| --- | --- |
| Date et signature | Date et signature |

**SOMMAIRE**

[**I.** **Composition de la base** 2](#_Toc222822551)

[**II.** **Table des variables** 2](#_Toc222822552)

1. **Table des variables**

| **Variables** | **Description** | **FORMAT** |
| --- | --- | --- |
| ID | Patient identifier | Texte |
| SEXE | Sex | 1="Male"  2="Female" |
| DDN | Date of birth | JJ/MM/AAAA |
| DDIAG | Date of Diagnosis | JJ/MM/AAAA |
| DATEV0 | Date of anti-TNFα initiation | JJ/MM/AAAA |
| AGEDIAG | Chronological age (y) | Numeric |
| AGEV0 | Age of anti-TNFα initiation | Numeric |
| AGEOSV0 | Bone age (y) | Numeric |
| DELAIDIAGBIO | Disease duration (y) | Numeric |
| TOPOL2 | Disease location | 1="L3/L3+L4/L1+L4"  2="L2/L2+L4" |
| TYPEB | Disease behavior | 1="B1"  2="B2"  3="B1/B2" |
| PERINA | Perianal disease | 1="Yes" 0="No" |
| MANDIGE | Extradigestive symptoms | 1="Yes" 0="No" |
| SCO | Harvey-Bradshaw Index | 0="[0-4["  1="[4-12["  2=" > = 12" |
| AMISALIV0 | 5 aminosalicylates | 1="Yes" 0="No" |
| CORTICOV0 | Corticosteroids | 1="Yes" 0="No" |
| BUDEV0 | Budesonide | 1="Yes" 0="No" |
| IMUNOV0 | Immunomodulator | 1="Yes" 0="No" |
| NEDCV0 | Enteral nutrition | 1="Yes" 0="No" |
| VCV0 | Height velocity at anti-TNFα initiation (cm) | Numeric |
| TAILLV0 | Height at anti-TNFα initiation (cm) | Numeric |
| IMCV0 | Body mass index at anti-TNFα initiation (kg/m^2^) | Numeric |
| VCV0SDS | Height velocity at anti-TNFα initiation (standard deviation score) | Numeric |
| TAILLV0SDS | Height standard deviation score at anti-TNFα at initiation | Numeric |
| IMCV0SDS | Body mass index at anti-TNFα initiation (standard deviation score) | Numeric |
| ALBUV0 | Albumin (g/L) | Numeric |
| CRPV0 | CRP (mg/L) | Numeric |
| TAILLDIAGSDS | Height at diagnosis (standard deviation score) | Numeric |
| IMCDIAGSDS | Body mass index at diagnosis (standard deviation score) | Numeric |
| TAILLCIBG | Target height (cm) | Numeric |
| TAILLCIBGSDS | Target height (standard deviation score) | Numeric |
| VCSDS1AN | Height velocity ( standard deviation score) during the 1^st^ year of anti-TNFα therapy | Numeric |
| VCSDS2ANS | Height velocity (standard deviation score) during the 2^nd^ year of anti-TNFα therapy | Numeric |
| VCSDS3ANS | Height velocity standard deviation score during the 3rd year of anti-TNFα therapy | Numeric |
| ALBUV1AN | Albumin (g/L) at year 1 of anti-TNFα therapy | Numeric |
| CRPV1AN | CRP (mg/L) at year1 of anti-TNFα therapy | Numeric |
| AGEVIS | Age at the end of anti-TNFα therapy | Numeric |
| DUREETTT | Duration of treatment (years) | Numeric |
| SCOV | Harvey-Bradshaw Index at adult height or last follow up | 0="[0-4["  1="[4-12["  2=" > = 12" |
| AMISALIV | 5 aminosalicylates at adult height or last follow up | 1="Yes" 0="No" |
| CORTICOV | Corticosteroids at adult height or last follow up | 1="Yes" 0="No" |
| BUDEV | Budesonide at adult height or last follow up | 1="Yes" 0="No" |
| IMUNOV | Immunomodulator at adult height or last follow up | 1="Yes" 0="No" |
| NEDCV | Enteral nutrition at adult height or last follow up | 1="Yes" 0="No" |
| VC | Height velocity in the year preceding the attainment of adult height or last follow up (cm) | Numeric |
| TAILLV | Adult height or last follow up (cm) | Numeric |
| IMCV | Body mass index at adult height or last follow up | Numeric |
| VCSDS | Height velocity in the year preceding the attainment of adult height or last follow up (standard deviation score) | Numeric |
| TAILLVSDS | Adult height (standard deviation score) | Numeric |
| IMCVSDS | Body mass index at adult height or last follow up (standard deviation score) | Numeric |
| ALBUV | Albumin (g/L) at adult height or last follow up | Numeric |
| CRPV | CRP (mg/L) at adult height or last follow up | Numeric |
| TAILLMAX | Adult height | 1="Yes" 0="No" |
