# Supplementary material for: Growth and Adult Height in Patients with Crohn's Disease Treated with Anti-Tumor Necrosis Factor α Antibodies
Source: PLoS One. 2016 Sep 16;11(9):e0163126. doi: 10.1371/journal.pone.0163126 (PMC5026336; doi:10.1371/journal.pone.0163126)
Supplement: S1 Table — (DOCX) [file pone.0163126.s002.docx]

**S1 Table. Patients‘ treatments at anti-TNFα initiation.**

| Treatments | Patients (n=61) |
| --- | --- |
| No treatment | 8 |
| 5 aminosalicylates (5 asa) | 1 |
| Corticosteroids | 4 |
| Budesonide | 2 |
| Immunomodulator | 8 |
| Enteral Nutrition (EN) | 1 |
| Corticosteroids+Immunomodulator | 21 |
| Corticosteroids+Immunomodulator+5 asa | 3 |
| Corticosteroids+Immunomodulator+EN | 2 |
| Corticosteroids+Immunomodulator+Budesonide | 1 |
| Immunomodulator+Budesonide | 2 |
| Immunomodulator+5 asa | 2 |
| Immunomodulator+EN | 4 |
| 5 asa+Corticosteroids | 1 |
| 5 asa+Budesonide+EN | 1 |
