# Supplementary material for: Growth and Adult Height in Patients with Crohn's Disease Treated with Anti-Tumor Necrosis Factor α Antibodies
Source: PLoS One. 2016 Sep 16;11(9):e0163126. doi: 10.1371/journal.pone.0163126 (PMC5026336; doi:10.1371/journal.pone.0163126)
Supplement: S2 Table — (DOCX) [file pone.0163126.s003.docx]

**S2 Table. Reasons for starting anti -TNFα therapy**

|  | Patients (n) |
| --- | --- |
| Relapses despite immunosuppressive therapy | 17 |
| Corticosteroid dependence | 13 |
| Perianal disease | 6 |
| Contra-indications to immunosuppressive drugs | 4 |
| Relapses despite immunosuppressive therapy  and corticosteroid dependence | 12 |
| Relapses despite immunosuppressive therapy  and perianal disease | 4 |
| Relapses despite immunosuppressive therapy  and contra-indications to immunosuppressive drugs | 5 |
